# Supplementary material for: Affinity-enhanced RNA-binding domains as tools to understand RNA recognition
Source: Cell Rep Methods. 2023 Jun 26;3(6):100508. doi: 10.1016/j.crmeth.2023.100508 (PMC10326445; doi:10.1016/j.crmeth.2023.100508)
Supplement: Document S1. Figures S1–S4 [file mmc1.pdf]

Cell Reports Methods, Volume *3*

Supplemental information

Affinity-enhanced RNA-binding domains as tools to understand RNA recognition

Belén Chaves-Arquero, Katherine M. Collins, Giancarlo Abis, Geoff Kelly, Evangelos Christodoulou, Ian A. Taylor, and Andres Ramos

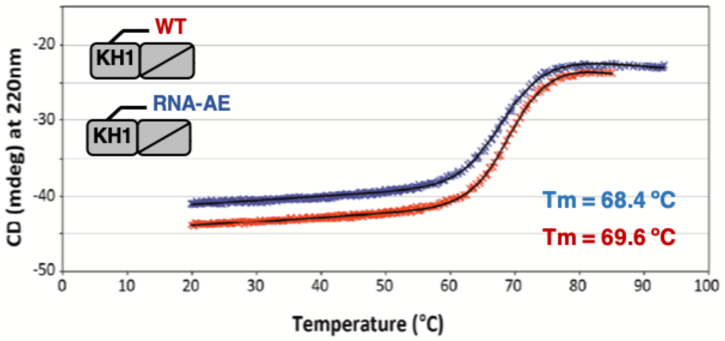

Figure S1: Stability of the affinity-enhanced FMRP KH12 GKKG mutant, related to Figure 1. Comparison of the thermal unfolding of FMRP KH12 WT (red) and RNA-AE (blue) monitored at 220 nm against temperature. Tm values are 69.9 ºC and 68.4 ºC respectively.

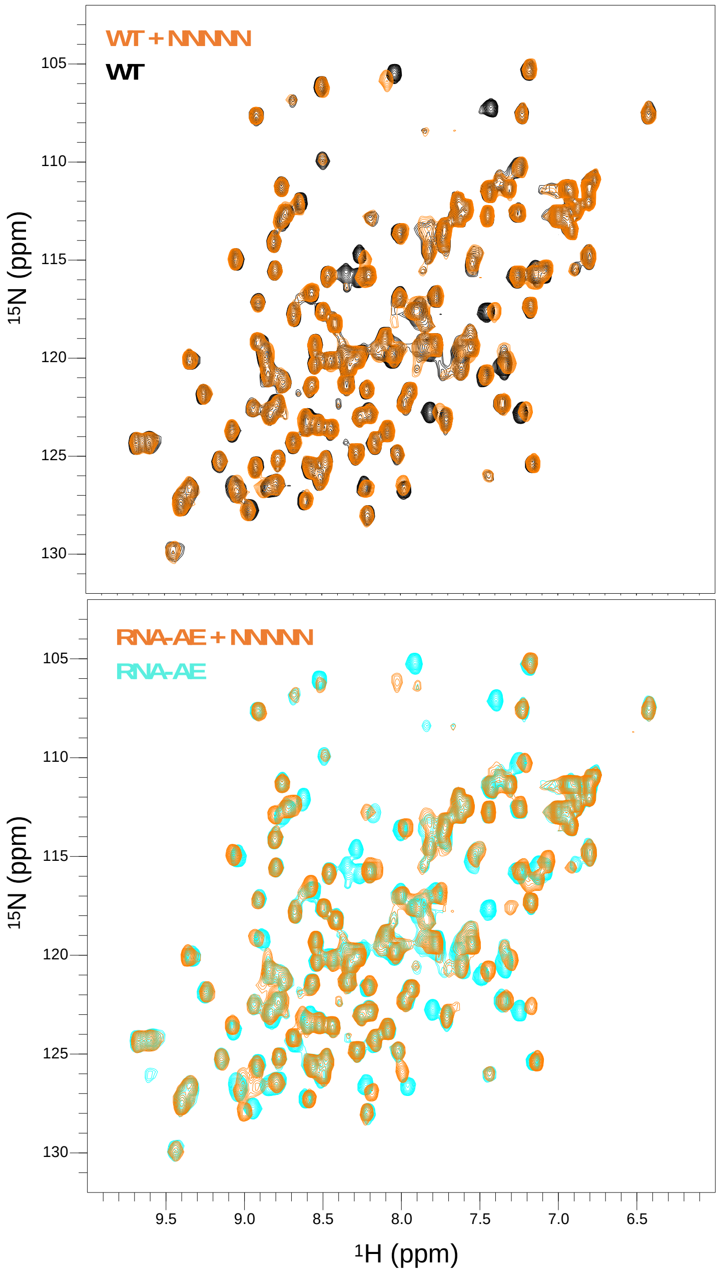

Figure S2: **RNA binding by the affinity-enhanced FMRP KH12 GKKG mutant, related to Figure 1.** Top: superimposition of ~1 hour ^{1}H-^{15}N HSQC spectra recorded on 50 µM FMRP WT free (black) and with NNNNN RNA at a 1:8 protein:RNA ratio (orange). Bottom: the equivalent superimposition is plotted for the spectra of FMRP RNA-AE free (light blue) and with NNNNN RNA at 1:8 protein:RNA ratio (orange).

A)

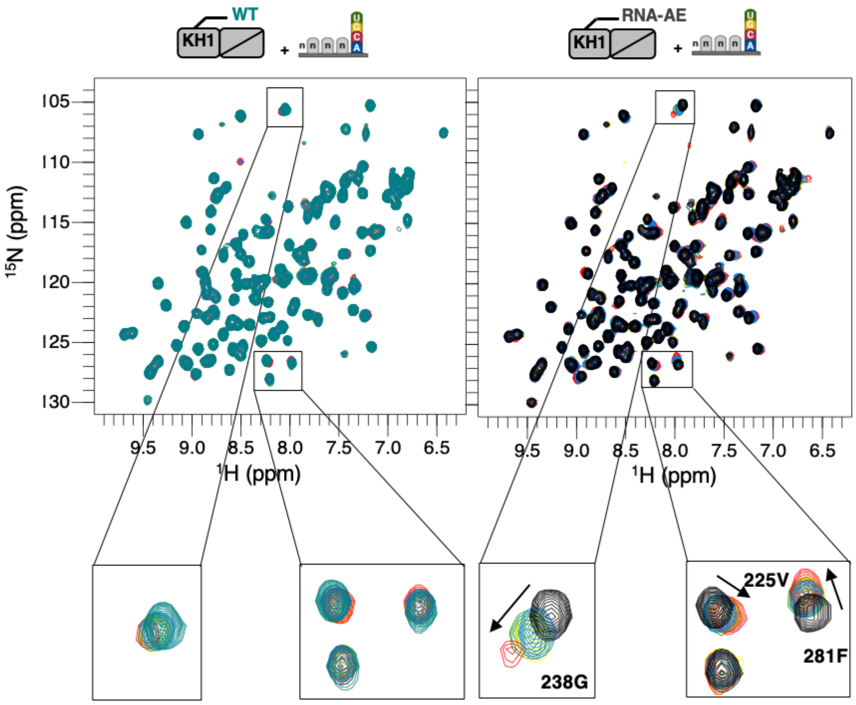

B)

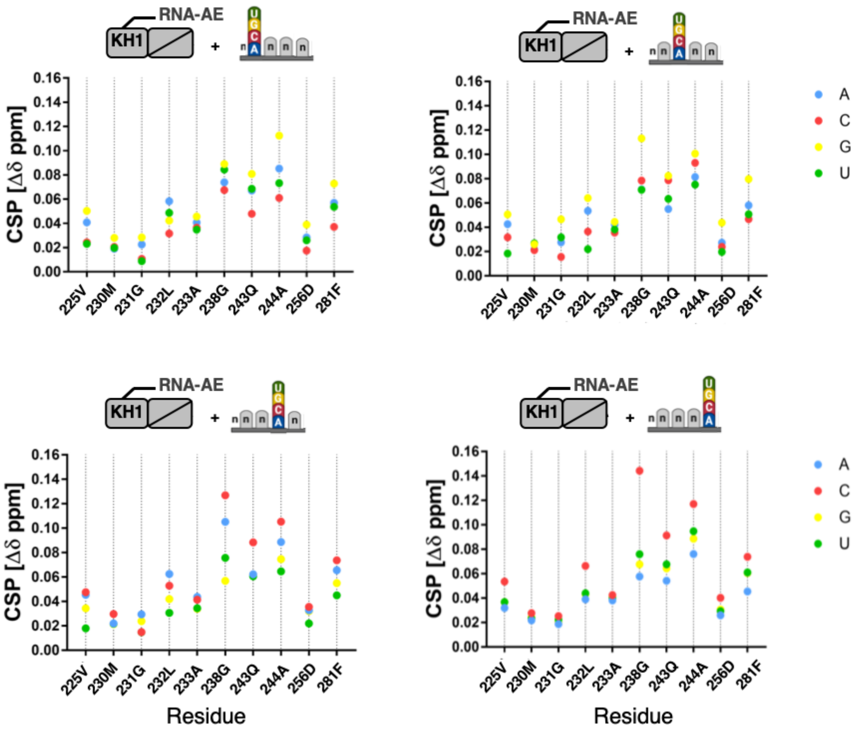

C)

	n X n n n	n n X n n	n n n X n	n n n n X
A	0.81	0.75	0.89	0.65
C	0.58	0.68	0.93	1.00
G	0.97	1.00	0.72	0.75
U	0.69	0.65	0.60	0.76

Figure S3. **Scaffold Independent Analysis (SIA) of the affinity-enhanced FMRP KH12 GKKG mutant, related to Figure 2.** The figure includes the Scaffold Independent Analysis (SIA) workflow, example data, and final scores. The SIA analysis provides a semi-quantitative, comparative estimate of the nucleobase preference of an RNA binding domain for each of the position of the bound oligonucleotide. (A) Superimposition of the ^{1}H-^{15}N HSQC spectra for the FMRP WT (left) and FMRP RNA-AE (right) free and when bound to each of the four quasi-randomized RNA pools which differ in the nucleobase to be examined (either A, C, G or U), for position 4 in this example. These superimpositions are used to measure the shift in peak position that report on the binding affinity of the four pools. Zoomed regions are for better comparison of the magnitude of the shifts observed in the 238G, 225V and 281F residues in both FMRP WT and FMRP RNA-AE spectra. The zoom-ins show that, while shifts in the wild type protein are very small, at the limit of the spectral resolution, shifts in the mutant can be measured with confidence and clear differences are visible. (B) Chemical shift changes (weighted average of ^{1}H and ^{15}N) of individual resonances of FMRP RNA-AE when binding to the four quasi-randomized RNA pools. For each peak, the values reported are normalized with respect to the highest shift value so that each peak contributes equally to the output. This is to ensure that any local difference due to the chemical nature of the bases to be scanned is more robustly averaged out across all the peaks, yielding final SIA scores depending on affinity. (C) Final SIA scores for the four positions.

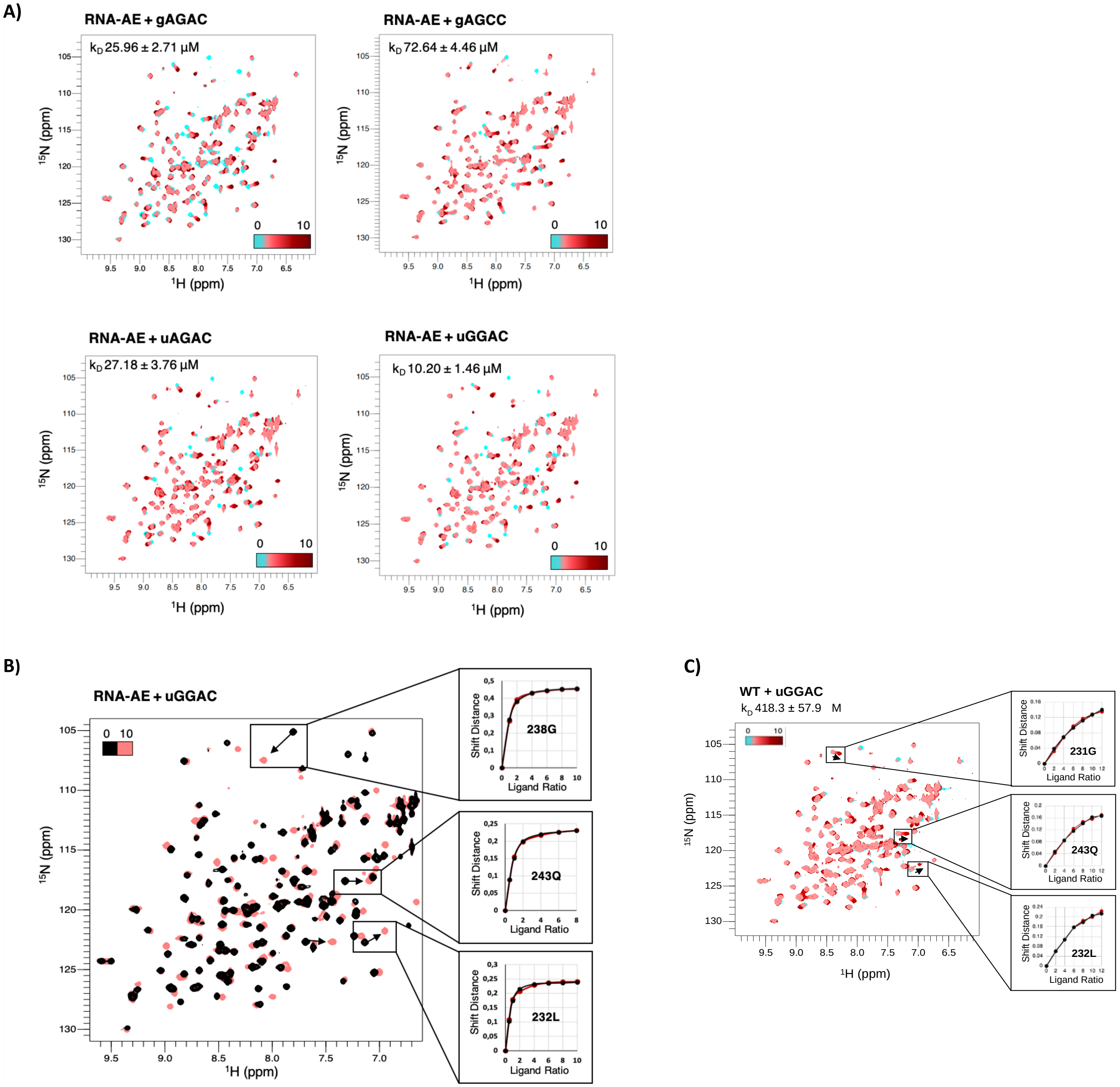

A)
RNA-AE + gAGAC
kD 25.96 ± 2.71 μM
RNA-AE + gAGCC
kD 72.64 ± 4.46 μM
RNA-AE + uAGAC
kD 27.18 ± 3.76 μM
RNA-AE + uGGAC
kD 10.20 ± 1.46 μM
0
10
15N (ppm)
1H (ppm)
B)
RNA-AE + uGGAC
0 10
15N (ppm)
1H (ppm)
Shift Distance
Ligand Ratio
238G
243Q
232L
C)
WT + uGGAC
kD 418.3 ± 57.9 M
15N (ppm)
1H (ppm)
Shift Distance
Ligand Ratio
231G
243Q
232L

Figure S4: **Affinity of FMRP KH12 wild type and affinity-enhanced for the specific sequences related to Figure 3.** RNA recognition by FMRP RNA-AE. (A) Overlaid ^{1}H-^{15}N HSQC spectra of FMRP RNA-AE with gAGAC, gAGCC, uAGAC and uGGAC at protein to RNA ratios of 1 to 0, 0.5, 1, 2, 4, 6, 8, 10. The spectra are coloured with increasingly dark red as we proceed along the titration. (B) Overlaid ^{1}H-^{15}N HSQC spectra of FMRP RNA-AE with uGGAC at protein to RNA ratio 1 to 10. The binding isotherm derived from the fit of chemical shift change against protein : RNA ratio is reported for three representative peaks, which are also displayed. Data points are coloured red, while the fit is in black. Fitting was performed using CcpNmr and the equation A(B+x-sqrt((B+x)^2-4x. Overall K_D values reported in the manuscript are an average of the ones obtained for the individual residues, and errors are reported. (C) Overlaid ^{1}H-^{15}N HSQC spectra of FMRP WT recorded during a titration with the preferred "uGGAC" sequence at protein-to-RNA ratios of 1 to 0, 0.5, 1, 2, 4, 6, 8, 10. The spectra are coloured with increasingly dark red as we proceed along the titration. The binding isotherms derived from the fit of chemical shift changes against protein:RNA ratios are reported for three representative peaks, the same used for the GKKG mutant in Figure S4b. In this plots, experimental data are in red, while the fit is in black. Fitting was performed using CcpNmr and the equation A(B+x-sqrt((B+x)^2-4x. The average K_D value and the errors are reported.
