# Supplementary material for: Affinity-enhanced RNA-binding domains as tools to understand RNA recognition
Source: Cell Rep Methods. 2023 Jun 26;3(6):100508. doi: 10.1016/j.crmeth.2023.100508 (PMC10326445; doi:10.1016/j.crmeth.2023.100508)
Supplement: Document S2. Article plus supplemental information [file mmc2.pdf]

Cell Reports Methods

Report

Affinity-enhanced RNA-binding domains as tools to understand RNA recognition

Graphical abstract

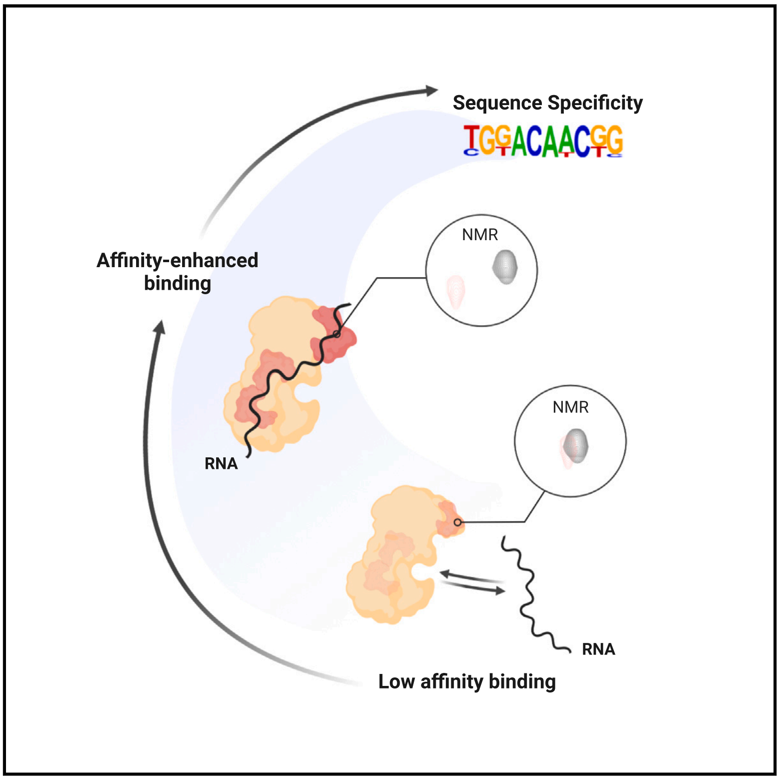

Highlights

- Specificity and function of low-affinity RNA-binding domains are often unclear
- We propose a strategy to increase domain affinity but maintain specificity
- We use it to reveal how a domain of the FRX protein recognizes RNA targets

Authors

Belén Chaves-Arquero, Katherine M. Collins, Giancarlo Abis, Geoff Kelly, Evangelos Christodoulou, Ian A. Taylor, Andres Ramos

Correspondence

a.ramos@ucl.ac.uk

In brief

Low-affinity domains are common in RNA-binding proteins, but their sequence specificity and function are poorly understood. Chaves-Arquero et al. propose enhancing the domains' affinity to better define their specificity. An NMR-based strategy is applied to the FXR protein, revealing a role for a low-affinity RNA-binding domain in RNA target selectivity.

Chaves-Arquero et al., 2023, Cell Reports Methods *3*, 100508
June 26, 2023 © 2023 The Authors.
https://doi.org/10.1016/j.crmeth.2023.100508

CellPress

Cell Reports Methods

CellPress
OPEN ACCESS

Report

Affinity-enhanced RNA-binding domains as tools to understand RNA recognition

Belén Chaves-Arquero,[1,2,6] Katherine M. Collins,[1,6] Giancarlo Abis,[1] Geoff Kelly,[3] Evangelos Christodoulou,[4] Ian A. Taylor,[5] and Andres Ramos[1,7,*]
[1]Institute of Structural and Molecular Biology (ISMB), University College London, London WC1E 6AA, UK
[2]Department of Structural and Chemical Biology, Center for Biological Research, CIB, CSIC, Av. Ramiro de Maeztu 9, 28040 Madrid, Spain
[3]The Medical Research Council Biomedical NMR Centre, the Francis Crick Institute, 1 Midland Road, London NW1 1AT, UK
[4]Structural Biology Science Technology Platform, the Francis Crick Institute, 1 Midland Road, London NW1 1AT, UK
[5]Macromolecular Structure Laboratory, the Francis Crick Institute, 1 Midland Road, London NW1 1AT, UK
[6]These authors contributed equally
[7]Lead contact
*Correspondence: a.ramos@ucl.ac.uk
https://doi.org/10.1016/j.crmeth.2023.100508

MOTIVATION RNA-binding domains represent the building blocks of RNA regulation, and their sophisticated nucleic acid recognition builds into the selectivity and functional variation of complex post-transcriptional regulatory networks. The RNA recognition properties and the function of some domains are well characterized. However, as current methods to dissect RNA recognition require the interaction to be relatively stable, the low affinity of many other domains prevents the characterization of their RNA recognition properties and the understanding of the role they play in protein function. To overcome this limitation, we propose to use protein design to enhance a domain's affinity of RNA binding without altering nucleobase recognition and specificity. We use these affinity-enhanced domains to reveal their RNA sequence specificity and their role in target selection in the cell and protein function.

SUMMARY

Understanding how the RNA-binding domains of a protein regulator are used to recognize its RNA targets is a key problem in RNA biology, but RNA-binding domains with very low affinity do not perform well in the methods currently available to characterize protein-RNA interactions. Here, we propose to use conservative mutations that enhance the affinity of RNA-binding domains to overcome this limitation. As a proof of principle, we have designed and validated an affinity-enhanced K-homology (KH) domain mutant of the fragile X syndrome protein FMRP, a key regulator of neuronal development, and used this mutant to determine the domain's sequence preference and to explain FMRP recognition of specific RNA motifs in the cell. Our results validate our concept and our nuclear magnetic resonance (NMR)-based workflow. While effective mutant design requires an understanding of the underlying principles of RNA recognition by the relevant domain type, we expect the method will be used effectively in many RNA-binding domains.

INTRODUCTION

Post-transcriptional RNA regulation expands genomic diversity and is key to cellular differentiation and organismal development. Understanding how RNA-binding proteins recognize the RNA targets is a key step to rationalize the selectivity of the RNA regulatory networks.[1,2] In the last decade, the extensive use of methods that map the interaction of proteins with cellular RNAs has provided an overview of the protein-RNA-binding landscape.[3–5] However, in many cases, our molecular understanding of protein-RNA interactions is far from complete. This is, at least in part, because we lack the molecular models of recognition required to interpret the interactions in the cell. In particular, we require information on the sequence specificity of the low-affinity RNA-binding protein domains that are common within the multi-domain regulators. Understanding how these regulators select the RNA targets requires an insight into the sequence specificity and affinity of all of the domains.[1,2] The RNA-binding specificity of a protein can be examined using a range of *in vitro* methodologies (reviewed in Dasti et al.[6]). However, the analysis can be a challenge for domains that bind RNA with low affinity, as most current methodologies are optimized for stable interactions. A number of these domains are outside the range of current methods, even those aimed at low-affinity

This is an open access article under the CC BY license (http://creativecommons.org/licenses/by/4.0/).

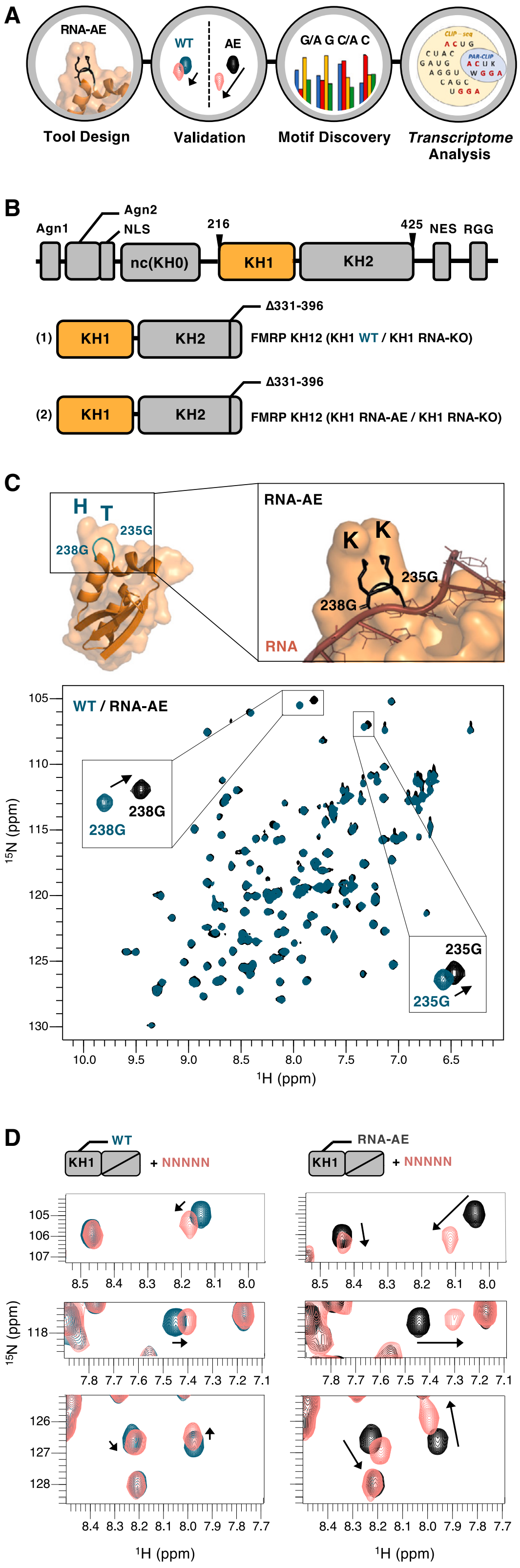

interactions, such as scaffold-independent analysis (SIA).[7] As a consequence, low-affinity RNA-binding domains are often reported to be non-specific and assumed to provide a limited contribution to target selection. In order to define the specificity of the domain and test the contribution to RNA target selection, we propose an orthogonal approach, i.e., to increase the affinity of weakly interacting RNA-binding domains and bring them within the useful range of existing methods.

In many well-studied systems, increasing the affinity of a macromolecular interaction without significantly changing the binding mode can be challenging. However, in low-affinity RNA-binding domains, including many K-homology (KH), RNA recognition motif (RRM), and zinc-finger (ZnF) domains, protein-RNA contacts are often not optimized to the same extent, and there is scope to enhance the strength of the interaction. Here, we use the KH1 domain of the fragile X syndrome protein FMRP as a paradigm for a class of low-affinity RNA-binding domains whose contribution to RNA recognition is unclear. We have designed a structure-based, localized mutation that increases KH1 RNA-binding affinity without affecting its RNA-binding mode. Then, we have shown how this mutation can be used to define the domain's sequence specificity and to rationalize FMRP *in vivo* recognition of its target sequences. This work both provides a proof of principle for the use of an RNA affinity-enhancing (AE) mutational strategy in the molecular investigation of target selectivity by RNA-binding proteins and defines a nuclear magnetic resonance (NMR) toolbox to execute this strategy (Figure 1A). It also provides an important molecular insight into a key neuronal regulator and a mutation/tool that could be used by the FMRP community in a range of *in vivo* studies to probe the role of KH1.

FMRP is a multi-domain RNA-binding protein essential for the correct development and function of the brain. FMRP misexpression, or a dysfunctional mutant protein, is the causal factor of fragile X syndrome, the most common inherited form of cognitive impairment.[9] Because of its pivotal role in the development of the nervous system and its medical relevance, FMRP has been extensively studied at the molecular, cellular, and systemic level in mammals, in model systems, and *in vitro*. These studies

Figure 1. The RNA-AE strategy and the KH1 RNA-AE mutant

(A) Workflow (left to right) and representative data for this RNA-AE concept.

(B) Top: domain structure of hFMRP—including the two Agenet domains, the non-canonical KH0 domain, the KH12 di-domain and carboxy-terminal, a nuclear export signal, and the low-complexity RGG region—that recognize G-quartet RNA elements. Here, we have used a previously characterized hFMRP construct that comprises the KH1 and KH2 domains minus the expanded loop of KH2 characterized in earlier studies[8] (amino acids 216–425 minus a 331–396 deletion). Bottom: a well-studied mutation that eliminates RNA binding (RNA-KO) has been inserted in KH2. (1) represents the wild-type (WT) KH1 version and (2) the RNA-AE, KK-mutated version of this construct.

(C) Top: RNA-AE mutant of FMRP KH1. Protein backbone is shown as a cartoon, and the two lysine side chains introduced by the KK mutation are modeled between 238G and 235G in the loop. Bottom: overlaid ^{1}H-^{15}N heteronuclear single quantum coherence (HSQC) spectra of 50 μM FMRP WT (teal) and 50 μM FMRP RNA-AE (black).

(D) Overlaid ^{1}H-^{15}N HSQC spectra of (left) 50 μM FMRP WT (teal) with NNNNN RNA (salmon) at a 1:8 protein:RNA ratio and (right) FMRP RNA-AE (black) with NNNNN RNA (salmon) at the same ratio.

have linked FMRP function to RNA regulation at the molecular level, but our understanding of the process of RNA target selection is incomplete. FMRP contains multiple RNA-binding domains and recognizes both structured motifs, such as G-quartets, and single-stranded (ss) RNA elements.[9–11] However, how the protein would recognize the ssRNA targets is not understood.[9] FMRP displays, among others, two KH domains. KH domains typically recognize ssRNA, and the FMRP KH1 has been reported to bind homopolymeric ssRNA *in vitro.*[12] In addition, it has been shown that a mutation destabilizing the flanking KH2 domain, which makes contact with KH1, affects the recognition of ssRNA sequences in the cell,[13] and a construct comprising the KH1 and KH2 domains, plus additional flanking regions, binds selectively to an ssRNA sequence *in vitro.*[14] On the contrary, a recent biochemical study has reported that the KH1 and KH2 domains do not interact with ssRNA,[15] and structural studies have suggested they may instead bind to the ribosome.[16] The role of the KH domains in ssRNA recognition is unclear.

Notably, FMRP KH1 has been reported to bind ssRNA with low affinity.[12] The domain can be expressed as part of the KH1-2 structural unit,[17,18] and we reasoned that this offered us the opportunity to test our concept by providing physiologically relevant information on sequence specificity. It also exemplifies how the concept can be explored in those more "complex" cases where domains cannot be expressed individually. We first asked whether we could design a KH1 mutant with increased affinity while maintaining the domain's structure, stability, and RNA-binding mode. Then, we explored whether this mutant can be used to define the sequence specificity of KH1. Finally, we asked whether this specificity can be used to understand better the cellular protein-RNA interaction. Our results validate the concept and methods used in this paper and indicate how we can obtain important information on a key regulator of neuronal development. We finally discuss how to apply the strategy to different RNA-binding domains.

RESULTS AND DISCUSSION

In order to obtain a model system that allows examining the interaction between FMRP KH1 and RNA, we expressed KH1 within a KH1-2 di-domain, where KH2 has been mutated to eliminate the interaction with RNA, using a well-established RNA-knockout (KO) mutant[8] (Figure 1B). We refer to this construct as FMRP RNA wild type (RNA-WT), as the emphasis of this study is on the RNA-binding properties of KH1. Then, we used our general understanding of the KH-RNA interaction[19] to design an RNA-AE mutant. We mutated the KH1 GxxG loop, which interacts with the RNA backbone in KH domains and is flexible in the free protein,[19] from GTHG to GKKG, obtaining an RNA-AE/RNA-KO KH1-2 double mutant, which we refer to as FMRP RNA-AE (Figures 1B and 1C). Next, we used NMR as a multi-purpose tool to (1) test the structural conservation and the preservation of the RNA-binding mode in the mutant, (2) validate the increase in affinity, and (3) extract the specificity. The comparison of fingerprint NMR spectra of the KH1 WT and RNA-AE mutant constructs confirmed that the structure of the domain is maintained, with only very local changes being observed (Figure 1C). In addition, the circular dichroism (CD)-monitored unfolding curves of the KH1 WT and RNA-AE constructs indicate that the mutation has no significant effect on protein stability (Figure S1). We therefore proceeded to test whether the KK mutation increases the affinity of KH1 for ssRNA. In the absence of direct information on KH1 RNA sequence specificity, we titrated the fully randomized NNNNN RNA into either WT or mutated RNA-AE protein and recorded ^{15}N-correlation NMR experiments (Figures 1D and S2). The direction and size of the chemical shift changes across the protein spectrum indicated that, as originally proposed,[12] KH1 interacts with ssRNA. Further, the comparison of the changes in the protein spectra indicates that the affected peaks and the direction of the shift are the same—which indicates that WT and mutant have the same RNA-binding mode and, we expect, sequence preference. Importantly, the molar fraction of the bound protein (that we read as the distance traveled by the peak) is increased, indicating that the mutant has a higher affinity than the wild type (Figure 1D).

Then, we examined whether the mutation could help characterize the specificity of the domain using SIA as a step in our NMR workflow. SIA is an NMR-based method that allows defining the nucleobase preference of a domain in each of the positions of the bound RNA[7] (Figure 2A). The method is designed for protein-RNA interactions in the weak-intermediate range, which still excludes many RNA-binding domains of important regulators, which have K_D values in the sub-millimolar range. Briefly, NMR spectra are recorded on the domain free and when bound to quasi-degenerate RNA oligos with all but one randomized position. Comparison of the changes in the protein spectra when in complex with different oligos with either A, C, G, or U in a given sequence position reports on the nucleobase preference of the domain in that position. In practice, changes are measured as the chemical shift perturbations (CSPs) of backbone amide peaks that are in fast exchange on a chemical shift timescale. Normalization and averaging are then performed to obtain the final SIA scores[7] (Figures 2B and S3). We recorded SIA data for both the WT and RNA-AE KH1 constructs to assess whether the tighter binding of the mutant results in a meaningful improvement of the data. Although good-quality spectra were recorded for both protein constructs, the sizes of the chemical shift changes in the assays with the WT KH1 construct are too small to be measured accurately. Instead, the higher affinity of the mutant protein resulted in much larger chemical shift changes, and that allowed us to obtain reliable SIA scores for the four bound nucleobases examined (four nucleobases are recognized specifically in KH-RNA interactions[19]) (Figure 2B).

Our SIA data indicate that KH1 prefers a G and a C in positions 2 and 4, respectively. In positions 1 and 3, instead, G and A and A and C have similar scores, yielding a G/A-G-C/A-C sequence preference. Notably, SIA scores are semi-quantitative and comparative, and small differences depend on the choice of peaks and on small experimental variations.[7] In order to more precisely define the KH's sequence specificity, we tested the nucleobase preference of the domain in positions 1 and 3 by directly comparing the binding of the oligos/nucleobases with similar scores in our SIA table. For position 3,

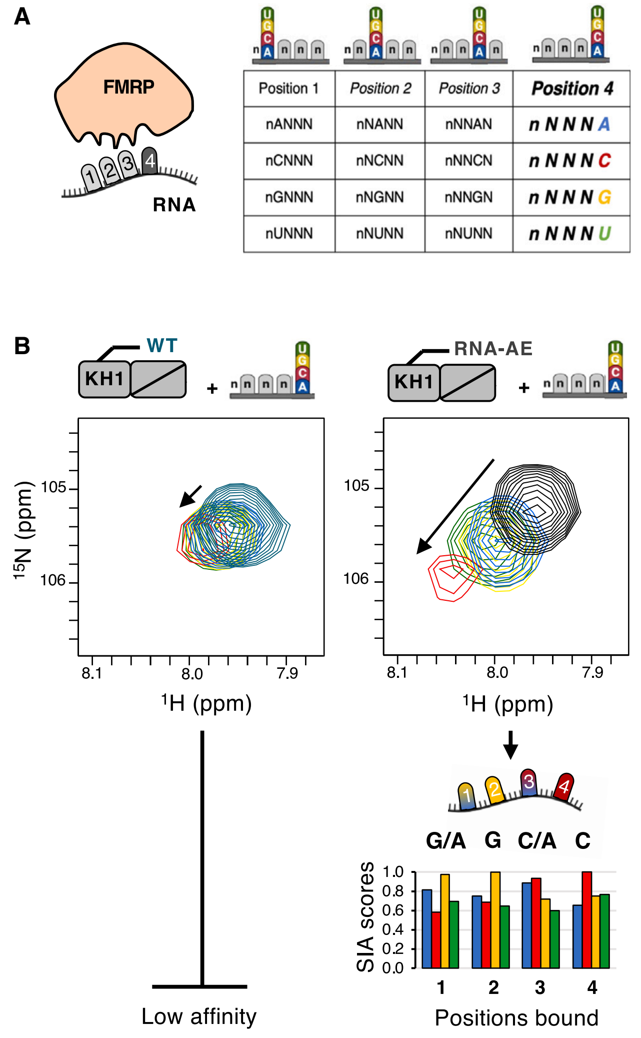

Figure 2. Scaffold-independent analysis (SIA) of FMRP RNA-AE

The data and workflow for the determination of the nucleobase preference of FMRP WT and FMRP RNA-AE for position 4 of the bound sequence are shown as an example.

(A) Four quasi-randomized RNA pools differing in the nucleobase to be examined (either A, C, G, or U) were added to the protein to a 1:4 ratio.

(B) ^{1}H-^{15}N HSQC spectra that were recorded for the free and bound proteins (FMRP WT and FMRP RNA-AE); a single peak is shown to highlight the differences in the shifts' magnitudes. The chemical shift changes were normalized with respect to the highest shift value so that each peak contributes equally to the output. Normalized values are averaged over the set of residues to give the final set of SIA scores.

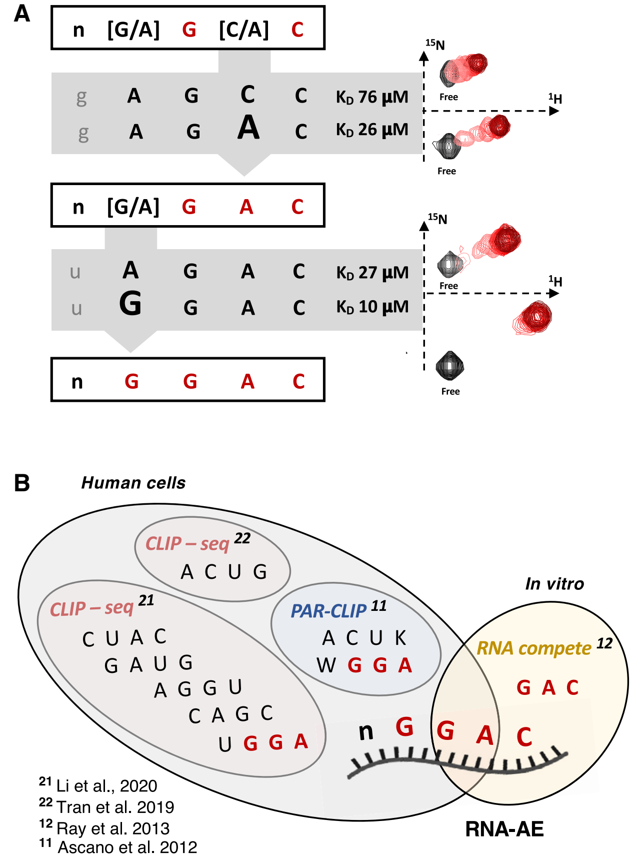

Figure 3. Refining the RNA-binding specificity of FMRP KH1

(A) The highest scoring oligos from the SIA pools (left) were tested using NMR (right). For the NMR data, a representative resonance is shown. The values of the equilibrium dissociation constant(s) obtained by fitting the change in peak position against the protein concentration are reported. Full spectra are shown in Figure S4.

(B) Comparison of the sequence identified in our RNA-AE approach with the motifs derived from in-cell (gray background) and *in vitro* (cream background) high-throughput assays on protein-RNA interactions.

we titrated the domain with the GAGCC and the GAGAC RNAs and measured the affinity of the two interactions by fitting the chemical shift changes in 2D ^{15}N-correlation NMR spectra against the protein/RNA ratio (Figures 3A and S4). This showed the protein prefers an A over a C with around 3-fold selectivity. We then examined whether the protein prefers an A or a G in position 1 by comparing the affinity of the UGGAC and UAGAC RNAs (Figures 3A and S4). Notably, while here we propose to use the mutant in SIA assays, depending on the affinity increase, it may be possible to test the specificity of the (mutated) domain using methods normally employed for higher affinities.

The result of our assays indicates that the domain recognizes a G with a 2.5-fold binding preference. Taken together, our SIA and follow-up assays define the domain's sequence preference as nGGAC. The recognition of specific ssRNA sequences is a significant but poorly understood element in FRMP selection of the cellular targets, and how FMRP recognizes these target sequences represents a key question in the biology of this protein. The most consistently found motif in the transcriptome-wide analysis of FMRP targets is GGA or WGGA (W = A/U), which was first identified in a PAR-CLIP (PhotoActivatable Ribonucleoside-enhanced CrossLinking and ImmunoPrecipitation) analysis[13] and later reported by others in cell analysis as UGGA.[9,20–22] To what extent this motif could be organized in G-quartet structures is debated, with some studies reporting an enrichment in patterns compatible with a G-quartet organization and others reporting distribution of

CellPress
OPEN ACCESS

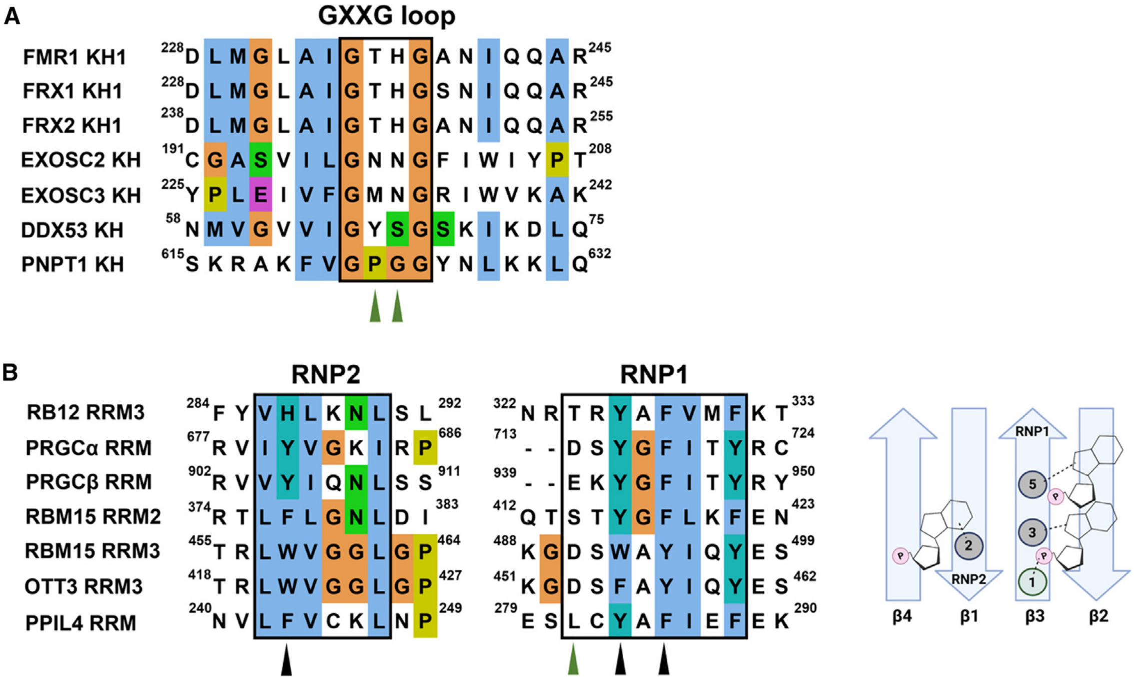

Figure 4. Application of RNA-AE concept and workflow to a range of KH and RRM domains

(A) Example KH domains with no positively charged residues in the GxxG loop found in a range of RNA regulators. The two green arrows indicate the two residues enclosed by the GxxG loop, which we propose could be mutated to lysine to enhance affinity.

(B) Left: an example set of RRM domains. We choose domains that are expected to bind single-stranded RNA in a canonical binding mode, as they contain three hallmark conserved hydrophobic residues (black arrows and right part of this panel). The green arrow indicates the position where a Lysine or an Arginine is found in high RNA-binding affinity RRM domains. We propose that, in the domains whose seqeunces are displayed, this residue could be mutated to Lys or Arg in order to obtain an affinity enhancement. Right: canonical RRM-RNA binding mode, the relevant contacts are represented.

motifs that do not reflect a G-quartet organization. Therefore, one important underlying question is which domain would recognize the GGA motif and whether the motif would be recognized by FMRP in a single-stranded background. Our strategy indicates that the KH1 domain recognizes the GGA sequence in an ssRNA setting (Figure 3B). Further, our work extends the recognized sequence to a GGAC tetranucleotide, which, in fact, includes the previously reported *in vitro*-recognized GAC sequence[14] (Figure 3B). This could imply that the multiple copies of the motif present in the target RNA sequence could mediate a multimerization of the protein on the RNA, the requirement for a high density of sites to increase the affinity, or both. Regardless and importantly, the higher affinity KH1 RNA-AE mutation, which increases binding affinity 40-fold (Figure S4), represents a tool to directly test the role of this domain in the binding to different RNA targets in the cell, and we expect that it will be useful to the broader community working on the role of FMRP in health and disease.

Our understanding of RNA recognition by common RNA-binding domains has increased thanks to high-resolution structures of protein-RNA complexes and to bioinformatic studies, and we now have detailed information on how domains such as KH, RRM, and many other common RNA-binding domains interact with the cognate RNAs. This information helps the design of AE mutants, extending the concept and NMR workflow we discuss here to RNA-binding domains from a range of RNA-binding proteins, and we expect that this will help us understand how these domains contribute to target recognition in the cell. For example, KH domains have a common RNA-binding mode, and in the free protein, the GxxG loop is exposed in solution.[19] The GKKG mutation used here can therefore be tested in KH domains with no positively charged residues in the GxxG loop (examples in Figure 4A) and can help define their specificity and contribution to target selection. Notably, AE mutations can be designed, in principle, in other common RNA-binding folds. For example, the interaction of the RRM domain, the most common RNA-binding domain, with the RNA targets has been studied in depth.[23] While RRMs can interact with RNA using different surfaces, the most common RNA-binding mode involves the binding of three hallmark aromatics in the domain β-sheet to the RNA bases.[23] In addition, a charged residue in position 1 is often found to make contact with the RNA backbone, and its mutation leads to a strong decrease in RNA affinity.[24] Notably, in a number of RRMs where the sequence specificity is unclear, this position is not occupied by a positively charged amino acid (examples in Figure 4B), and mutating the relevant amino acid to a lysine or arginine in this position would represent an effective AE strategy. Importantly, the method is not meant to be high throughput but rather is meant to be applied to the specific domains of interest to obtain important information on the domain RNA recognition and function. The potential of the approach is testified by the insight we have gained from the analysis of the

fragile X protein, a system important but challenging to study. Our results answer an important question about that system—how does the protein FMRP recognize ssRNA sequences—and provide a working model and a tool (a mutant) to explore this concept further in functional studies.

Limitations of the study

In order to design AE mutation(s), an understanding of the RNA-binding mode of each domain is required, and it is possible that a number of mutants need to be tested, depending on the domain to be examined. For KH domains, we expect that it will be generally possible to use the GxxG to GKKG mutation in the investigation as (1) KH domains have a common RNA-binding mode and (2) the GxxG loop is surface exposed and can be mutated without affecting the domain's fold or stability. For other domains, where one or more structures in complex with RNA are available, we expect it will be possible to identify amino acids that are positioned in proximity of nucleic acid backbone phosphates that could be mutated to positively charged residues in homologs to optimize affinity without affecting nucleobase recognition. We use the well-studied RMM domain to exemplify this design. However, if no structural information is available, for example for newly identified RNA-binding domains, then designing AE mutants is likely to be quite difficult. It is also important to point out that while the mutants are valuable tools to understand target recognition and protein function, mutant design and testing is time demanding. Therefore, rather than high throughput, the concept we propose is to focus the experimental workflow on a specific protein of interest, which can be then studied *in vivo*.

From a more technical stance, the SIA experiments require ^{15}N labeling of the protein domain. While straightforward with proteins expressed recombinantly in *Escherichia coli*, this is more complex in other expression systems. Additionally, there is a requirement for the protein to be stable at micromolar concentration for a period of days. This is typically not a problem for proteins that have been previously studied with biochemical or biophysical methods, and a range of buffer and experimental parameters can be used to help with this. Finally, as NMR is a size-sensitive method, experiments will typically provide better data for smaller domains or proteins. While in a majority of cases RNA-binding domains are typically of a size amenable to NMR studies (8–15 kDa), in isolation or as di- or even tri-domains, size should be considered.

STAR★METHODS

Detailed methods are provided in the online version of this paper and include the following:

- KEY RESOURCES TABLE
- RESOURCE AVAILABILITY
  - Lead contact
  - Materials availability
  - Data and code availability
- EXPERIMENTAL MODEL AND SUBJECT DETAILS
  - *In vitro* studies
- METHOD DETAILS
  - Cloning and mutagenesis
  - Protein expression and purification
  - RNA sample preparation
  - NMR experiments
  - Scaffold independent analysis (SIA)
  - NMR binding assays
  - Thermal unfolding by circular dichroism (CD)
  - Sequence alignments
- QUANTIFICATION AND STATISTICAL ANALYSIS

SUPPLEMENTAL INFORMATION

Supplemental information can be found online at https://doi.org/10.1016/j.crmeth.2023.100508.

ACKNOWLEDGMENTS

This work was supported by the UK Medical Research Council (MC_PC_13051, U117574558, and MR/S000305/1) and the UK BBRSC research grant S014438/1. It was also supported by University College London and by the Francis Crick Institute, which receives its core funding from Cancer Research UK (CC2029), the UK Medical Research Council (CC2029), and the Wellcome Trust (CC2029). NMR spectra were recorded at the MRC Biomedical NMR Facility Francis Crick Institute UK, which is funded by Cancer Research UK (CC1078), the UK Medical Research Council (CC1078), and the Wellcome Trust (CC1078) and at UCL NMR facilities. We thank Alain Oregioni and Angelo Pinto De Figueiredo for assistance. We also thank Abigail Turner for assistance in the bioinformatic analysis.

AUTHOR CONTRIBUTIONS

K.M.C. and E.C. cloned the required constructs. K.M.C. and B.C.-A. purified the required proteins. B.-C.A., K.M.C., G.A., and G.K. recorded and analyzed the NMR experiments. A.R., B.C.-A., G.A., and I.A.T. wrote the paper together with the other authors.

DECLARATION OF INTERESTS

The authors declare no competing interests.

Received: October 18, 2022
Revised: March 14, 2023
Accepted: May 30, 2023
Published: June 26, 2023

REFERENCES

1. Lunde, B.M., Moore, C., and Varani, G. (2007). RNA-binding proteins: modular design for efficient function. Nat. Rev. Mol. Cell Biol. *8*, 479–490.
2. Hennig, J., and Sattler, M. (2015). Deciphering the protein-RNA recognition code: combining large-scale quantitative methods with structural biology. Bioessays *37*, 899–908.
3. Sugimoto, Y., Vigilante, A., Darbo, E., Zirra, A., Militti, C., D'Ambrogio, A., Luscombe, N.M., and Ule, J. (2015). HiCLIP reveals the in vivo atlas of mRNA secondary structures recognized by Staufen 1. Nature *519*, 491–494.
4. Wheeler, E.C., Van Nostrand, E.L., and Yeo, G.W. (2018). Advances and challenges in the detection of transcriptome-wide protein–RNA interactions. Wiley Interdiscip. Rev. RNA *9*, e1436.
5. Licatalosi, D.D., and Darnell, R.B. (2010). RNA processing and its regulation: global insights into biological networks. Nat. Rev. Genet. *11*, 75–87.
6. Dasti, A., Cid-Samper, F., Bechara, E., and Tartaglia, G.G. (2020). RNA-centric approaches to study RNA-protein interactions in vitro and in silico. Methods *178*, 11–18.

7. Beuth, B., García-Mayoral, M.F., Taylor, I.A., and Ramos, A. (2007). Scaffold-independent analysis of RNA-protein interactions: the Nova-1 KH3-RNA complex. J. Am. Chem. Soc. *129*, 10205–10210.

8. Hollingworth, D., Candel, A.M., Nicastro, G., Martin, S.R., Briata, P., Gherzi, R., and Ramos, A. (2012). KH domains with impaired nucleic acid binding as a tool for functional analysis. Nucleic Acids Res. *40*, 6873–6886.

9. Richter, J.D., and Zhao, X. (2021). The molecular biology of FMRP: new insights into fragile X syndrome. Nat. Rev. Neurosci. *22*, 209–222.

10. Anderson, B.R., Chopra, P., Suhl, J.A., Warren, S.T., and Bassell, G.J. (2016). Identification of consensus binding sites clarifies FMRP binding determinants. Nucleic Acids Res. *44*, 6649–6659.

11. Goering, R., Hudish, L.I., Guzman, B.B., Raj, N., Bassell, G.J., Russ, H.A., Dominguez, D., and Taliaferro, J.M. (2020). FMRP promotes RNA localization to neuronal projections through interactions between its RGG domain and g-quadruplex RNA sequences. Elife *9*, e52621.

12. Adinolfi, S., Bagni, C., Musco, G., Gibson, T., Mazzarella, L., and Pastore, A. (1999). Dissecting FMR1, the protein responsible for fragile X syndrome, in its structural and functional domains. RNA *5*, 1248–1258.

13. Ascano, M., Mukherjee, N., Bandaru, P., Miller, J.B., Nusbaum, J.D., Corcoran, D.L., Langlois, C., Munschauer, M., Dewell, S., Hafner, M., et al. (2012). FMRP targets distinct mRNA sequence elements to regulate protein expression. Nature *492*, 382–386.

14. Ray, D., Kazan, H., Cook, K.B., Weirauch, M.T., Najafabadi, H.S., Li, X., Gueroussov, S., Albu, M., Zheng, H., Yang, A., et al. (2013). A compendium of RNA-binding motifs for decoding gene regulation. Nature *499*, 172–177.

15. Athar, Y.M., and Joseph, S. (2020). The human fragile X mental retardation protein inhibits the elongation step of translation through its RGG and C-terminal domains. Biochemistry *59*, 3813–3822.

16. Chen, E., Sharma, M.R., Shi, X., Agrawal, R.K., and Joseph, S. (2014). Fragile X mental retardation protein regulates translation by binding directly to the ribosome. Mol. Cell *54*, 407–417.

17. Musco, G., Kharrat, A., Stier, G., Fraternali, F., Gibson, T.J., Nilges, M., and Pastore, A. (1997). The solution structure of the first KH domain of FMR1, the protein responsible for the fragile X syndrome. Nat. Struct. Biol. *4*, 712–716.

18. Valverde, R., Pozdnyakova, I., Kajander, T., Venkatraman, J., and Regan, L. (2007). Fragile X mental retardation syndrome: structure of the KH1-KH2 domains of fragile X mental retardation protein. Structure *15*, 1090–1098.

19. Nicastro, G., Taylor, I.A., and Ramos, A. (2015). KH-RNA interactions: back in the groove. Curr. Opin. Struct. Biol. *30*, 63–70.

20. Suhl, J.A., Chopra, P., Anderson, B.R., Bassell, G.J., and Warren, S.T. (2014). Analysis of FMRP mRNA target datasets reveals highly associated mRNAs mediated by G-quadruplex structures formed via clustered WGGA sequences. Hum. Mol. Genet. *23*, 5479–5491.

21. Li, M., Shin, J., Risgaard, R.D., Parries, M.J., Wang, J., Chasman, D., Liu, S., Roy, S., Bhattacharyya, A., and Zhao, X. (2020). Identification of FMR1-regulated molecular networks in human neurodevelopment. Genome Res. *30*, 361–374.

22. Tran, S.S., Jun, H.-I., Bahn, J.H., Azghadi, A., Ramaswami, G., Van Nostrand, E.L., Nguyen, T.B., Hsiao, Y.H.E., Lee, C., Pratt, G.A., et al. (2019). Widespread RNA editing dysregulation in brains from autistic individuals. Nat. Neurosci. *22*, 25–36.

23. Maris, C., Dominguez, C., and Allain, F.H.T. (2005). The RNA recognition motif, a plastic RNA-binding platform to regulate post-transcriptional gene expression. FEBS *272*, 2118–2131.

24. Beusch, I., Barraud, P., Moursy, A., Cléry, A., and Allain, F.H.T. (2017). Tandem hnRNP A^{o} RNA recognition motifs act in concert to repress the splicing of survival motor neuron exon 7. Elife *6*, e25736.

25. Valverde, R., Edwards, L., and Regan, L. (2008). Structure and function of KH domains. FEBS *275*, 2712–2726.

26. Vranken, W.F., Boucher, W., Stevens, T.J., Fogh, R.H., Pajon, A., Llinas, M., Ulrich, E.L., Markley, J.L., Ionides, J., and Laue, E.D. (2005). The CCPN data model for NMR spectroscopy: development of a software pipeline. Proteins *59*, 687–696.

27. Sievers, F., Wilm, A., Dineen, D., Gibson, T.J., Karplus, K., Li, W., Lopez, R., McWilliam, H., Remmert, M., Söding, J., et al. (2011). Fast, scalable generation of high-quality protein multiple sequence alignments using Clustal Omega. Mol. Syst. Biol. *7*, 539.

28. Lee, W., Tonelli, M., and Markley, J.L. (2015). NMRFAM-SPARKY: enhanced software for biomolecular NMR spectroscopy. Bioinformatics *31*, 1325–1327.

29. Delaglio, F., Grzesiek, S., Vuister, G.W., Zhu, G., Pfeifer, J., and Bax, A. (1995). NMRPipe: a multidimensional spectral processing system based on UNIX pipes. J. Biomol. NMR *6*, 277–293.

30. Waterhouse, A.M., Procter, J.B., Martin, D.M.A., Clamp, M., and Barton, G.J. (2009). Jalview Version 2--a multiple sequence alignment editor and analysis workbench. Bioinformatics *25*, 1189–1191.

31. Mooij, W.T.M., Mitsiki, E., and Perrakis, A. (2009). ProteinCCD: enabling the design of protein truncation constructs for expression and crystallization experiments. Nucleic Acids Res. *37*, 402–405.

32. McIntosh, L.P., and Dahlquist, F.W. (1990). Biosynthetic incorporation of 15N and 13C for assignment and interpretation of nuclear magnetic resonance spectra of proteins. Q. Rev. Biophys. *23*, 1–38.

33. UK LAEMMLI (1970). Cleavage of structural proteins during the assembly of the head of bacteriophage T4. Nature *227*, 680–685.

34. Gasteiger, E., Hoogland, C., Gattiker, A., Duvaud, S., Wilkins, M.R., and Appel, R.D. (2005). The Proteomics Protocols Handbook (Springer), pp. 571–608.

35. Grzesiek, S., and Bax, A. (1992). An efficient experiment for sequential backbone assignment of medium-sized isotopically enriched proteins. J. Magn. Reson. *99*, 201–207.

36. Grzesiek, S., and Bax, A. (1992). Correlating backbone amide and side chain resonances in larger proteins by multiple relayed triple resonance NMR. J. Am. Chem. Soc. *114*, 6291–6293.

37. Johnson, B.A., and Blevins, R.A. (1994). NMR View: a computer program for the visualization and analysis of NMR data. J. Biomol. NMR *4*, 603–614.

38. Schanda, P., Kupce, E., and Brutscher, B. (2005). SOFAST-HMQC experiments for recording two-dimensional heteronuclear correlation spectra of proteins within a few seconds. J. Biomol. NMR *33*, 199–211.

39. Sjekloća, L., Konarev, P.V., Eccleston, J., Taylor, I.A., Svergun, D.I., and Pastore, A. (2009). A study of the ultrastructure of fragile-X-related proteins. Biochem. J. *419*, 347–357.

40. Paysan-Lafosse, T., Blum, M., Chuguransky, S., Grego, T., Pinto, B.L., Salazar, G.A., Bileschi, M.L., Bork, P., Bridge, A., Colwell, L., et al. (2023). InterPro in 2022. Nucleic Acids Res. *51*, D418–D427.

41. Goujon, M., McWilliam, H., Li, W., Valentin, F., Squizzato, S., Paern, J., and Lopez, R. (2010). A new bioinformatics analysis tools framework at EMBL-EBI. Nucleic Acids Res. *38*, W695–W699.

STAR★METHODS

KEY RESOURCES TABLE

REAGENT or RESOURCE	SOURCE	IDENTIFIER
**Bacterial and virus strains**		
Escherichia coli BL21(DE3)	New England Biolabs	C2527I
Escherichia coli BL21-Gold(DE3)	Agilent	200131
**Chemicals, peptides, and recombinant proteins**		
Dithiothreitol	Sigma-Aldrich	D0632
$^{15}NH_4Cl$	Merck Sigma-Aldrich	299251
Isopropyl ß-D-1-thiogalactopyranoside (IPTG)	*Neo* Biotech	NB-45-00030
Deuterium oxide	Merck Sigma Aldrich	756822
Lysozyme chloride form from chicken egg white	Merck Sigma-Aldrich	L-2971
DNAseI	Merck Sigma-Aldrich	10104159001
Protease inhibitors cOmplete™ EDTA-free ultra-tablets	Roche	05056489001
Sodium phosphate monobasic	Sigma Aldrich	S5011
Thiamine hydrochloride	SIGMA	T4625
Biotine	Merck Sigma	B4501
Tricine	Bio-Rad	1610713
HRV 3C protease	Home expressed	N/A
Protino® Ni-NTA agarose resin	Macherey-Nagel	745400.100
Imidazole, 99.5%	Sigma-Aldrich	56750
Sodium chloride, 99.5%	Fisher Scientific UK	S/3161/60
Potassium phosphate monobasic anhydrous	MP	195453
Yeast extract	Bio Basic	G0961
Tryptone	Bio Basic	TG217(G211)
Kanamycin sulfate	Sigma	K1377
Calcium chloride dihydrate	Sigma-Aldrich	C3881
Recombinant FMRP T236K/H237K into KH1 and K299D/N300D into KH2	This study	N/A
Recombinant FMRP K299D/N300D into KH2	This study	N/A
Agarose	VWR International	84609.0500
Sac1	New England Biolabs	R3156
Kpn1	New England Biolabs	R3142
T4 DNA polymerase	New England Biolabs	M0203
DpnI	New England Biolabs	R0176
KOD polymerase hot start master mix	Merck	71842
Magnesium sulfate	Sigma-Aldrich	208094
Sodium azide, ReagentPlus®, ≥99.5%	Sigma-Aldrich	S2002
RNasin® Ribonuclease Inhibitor	Promega	N251A
$^{13}C_6$-D-glucose, 99%	Cambridge Isotope Laboratories	CLM-1396
D-glucose, 99.5%	Sigma-Aldrich	G8270
TRIS-HCl	Severn Biotech Ltd	30-20-60
β-mercaptoethanol	Aldrich	M6250
Tris(2-carboxyethyl)phosphine hydrochloride (TCEP)	Lbp Bio	P1020
InstantBlue® Coomassie protein stain	Abcam	Ab119211
Invitrogen™ NuPAGE™ 4 to 12%, Bis-Tris, 1.0–1.5 mm precasted gels	Invitrogen	NP0322

(*Continued on next page*)

Continued

REAGENT or RESOURCE	SOURCE	IDENTIFIER
Invitrogen™ NuPAGE™ MES SDS running buffer (20X)	Invitrogen	NP0002
**Oligonucleotides**		
FMRP_FW cagggacccggtgcttcgcgtttccacgagcag	Sigma-Aldrich	N/A
FMRP_RV ggcaccagagcgttatttcaggtagttcagatgata	Sigma-Aldrich	N/A
FMRP_KH1_ T236K/H237K_FW gcttggctatcggcaa gaagggcgcgaatattca	Sigma-Aldrich	N/A
FMRP_KH1_ T236K/H237K_RV tgaatattcgcgcc cttcttgccgatagccaagc	Sigma-Aldrich	N/A
FMRP_KH1_ T236D/H237D_FW gcttggctatcggc gatgatggcgcgaatattcag	Sigma-Aldrich	N/A
FMRP_KH1_ T236D/H237D_RV ctgaatattcgcgc catcatcgccgatagccaagc	Sigma-Aldrich	N/A
FMRP_KH2_ K299D/N300D_FW ggtaaagtcattgg ggatgatggtaaacttatccagg	Sigma-Aldrich	N/A
FMRP_KH2_ K299D/N300D_RV cctggataagttt accatcatccccaatgactttacc	Sigma-Aldrich	N/A
Customized RNA oligonucleotides	Horizon Discovery	N/A
**Recombinant DNA**		
Plasmid: pET-47b	Addgene	71461–3
Modified gene encoding FMRP with the Δ331-396 deletion	This study	Valverde et al.[25]
**Software and algorithms**		
TopSpin 2.1 & TopSpin 3.5pL7	Bruker	https://www.bruker.com
PyMOL	Schödinger	https://pymol.org/
CcpNmr Analysis	Vranken et al.[26]	https://www.ccpn.ac.uk
Clustal Omega	Sievers et al.,[27]	https://www.ebi.ac.uk/Tools/msa/clustalo/
Python version 2.7	Python Software Foundation	https://www.python.org
BioRender	BioRender, Torontom ON, Canada	https://www.biorender.com/
Sparky	Lee et al.[28]	https://www.cgl.ucsf.edu/home/sparky/
NMRPipe and NMRDraw	Delaglio et al.[29]	https://www.ibbr.umd.edu/nmrpipe/index.html
Jalview	Waterhouse et al.[30]	https://www.jalview.org/
GraphPad Prism version 7.0	GraphPad Software, San Diego, California USA	https://www.graphpad.com
Excel Office	Microsoft, WA, USA	https://www.microsoft.com/en-gb/
**Other**		
NanoDrop™ 2000	Thermo Fisher Scientific	ND-2000
ÄKTA start purification system	Cytiva	29022094
HiLoad 16/600 Superdex 75 pg column	Cytiva	GE28-9893-33
New Brunswick Innova® 42 cooled shaking incubator	Eppendorf	M1335-0002
EC apparatus laboratory freeze drier	Modulyo	N/A
Megafuge 16R centrifuge	Thermo Scientific	75004271
BioSafe Avanti® J-26S XP high performance centrifuge	Beckman Coulter	B22984
JA-25.50 fixed-angle aluminum rotor	Beckman Coulter	363058
J-LITE JLA-8.1000 fixed-angle aluminum rotor	Beckman Coulter	363688
Ascend™ 800 MHz magnetic field	Bruker	N/A
Ultrashield™ 700 MHz magnetic field	Bruker	N/A
OXFORD 600 MHz	Oxford Instruments	N/A

(*Continued on next page*)

REAGENT or RESOURCE	SOURCE	IDENTIFIER
Continued		
SampleJet	Bruker	N/A
J-815 spectropolarimeter equipped with CDF-426S temperature-control system	Jasco	N/A
Shigemi 5 mm symmetrical NMR microtube assembly	Sigma-Aldrich	Z543349-1EA
SampleJet NMR tubes	Bruker	WIMWG30004SJ
PowerPac^{TM} Basic Power Supply	Bio-Rad	1645050
XCell SureLock^{TM} Mini-Cell	Invitrogen	EI0001
FMRP KH12 nuclear magnetic resonance assignment	BMRB	Accession number: 51606

RESOURCE AVAILABILITY

Lead contact

Further information and requests for resources and reagents should be directed to and will be fulfilled by the lead contact, Andres Ramos (a.ramos@ucl.ac.uk).

Materials availability

Plasmids generated in this study have been deposited to Addgene, pET-47b, 71461-3.

Data and code availability

- FMRP KH12 nuclear magnetic resonance assignment data have been deposited at the Biological Magnetic Resonance DataBank (BMRB) and are publicly available as of the date of publication. Accession number is listed in the key resources table.
- This paper does not report original code.
- Any additional information required to reanalyse the data reported in this paper is available from the lead contact upon request.

EXPERIMENTAL MODEL AND SUBJECT DETAILS

In vitro studies

Commercial *Escherichia coli* BL21(DE3) cells were used as the source organism to obtain the recombinant proteins studied in this work. The cells were stored at -80°C, and freshly transformed with the plasmids containing FMRP KH12, WT and RNA-AE coding sequences. Cell cultures and protein induction are detailed in the method details section.

METHOD DETAILS

Cloning and mutagenesis

A gene encoding FMRP with the Δ331-396 deletion,[25] codon-optimized for *E. coli* expression, was purchased from Eurofins. Primers were designed using the Crystallization Construct Designer on-line tool (https://ccd.rhpc.nki.nl)[31] and used to amplify the DNA region encoding KH1 and KH2 domains incorporating 5′ AND-3′ extensions complementary to sections of the vector to produce the inserts. The vector used was pET-47b which contains an N-terminal hexahistidine tag cleavable by Human Rhinovirus 3C protease and a resistance marker to kanamycin. The vector was digested with Kpn1/Sac1 and then both inserts and linearized vector were treated with T4 DNA polymerase (NEB) in the presence of dATP and dTTP respectively to produce complementary single stranded overhangs. The plasmid was transformed into BL21-Gold(DE3) (Agilent) using a standard heat shock protocol. In a second round of mutations, primers were designed to introduce the mutations T236D/H237D and T236K/H237K into KH1, and K299D/N300D into KH2 were introduced into the constructs by amplification of the plasmid using overlapping complementary primers with the mutation of interest inserted at the center of the oligonucleotides. Following PCR amplification parent DNA was removed by DpnI digestion. The primers used for cloning and mutagenesis are reported in the STAR protocol.

Protein expression and purification

The plasmid containing the FMRP KH12, WT and RNA-AE coding sequences was transformed in BL21(DE3) *E. coli* cells, which were used to inoculate 1000 mL of M9 minimal media containing $^{15}NH_4$ and ^{13}C-glucose as the only nitrogen and carbon sources

respectively, as previously described.[32] Cells were grown to an OD600 of 0.6 and protein expression was induced with Isopropyl β-*d*-1-thiogalactopyranoside at a final concentration of 0.5 mM. Cells were grown for a further 4 h at 37°C after induction, harvested by centrifugation, and cell pellets stored at −80°C.

Frozen cells were resuspended in equilibration buffer (10 mM Tris-HCl pH 8.0, 10 mM imidazole, 200 mM NaCl, 2 mM β-Mercaptoethanol with 0.01 mg/mL DNaseI (Sigma) and 200 μg/mL lysozyme (Sigma)), sonicated on ice and centrifuged at 17000 rpm for 1 h at 4°C. The recombinant protein was purified by immobilized metal ion affinity chromatography (IMAC), using Protino Ni-NTA Agarose resin (Macherey-Nagel) in a gravity-driven column. The resin was then washed with 10 CV of wash buffer (10 mM Tris-HCl pH 8.0, 10 mM imidazole, 1M NaCl, 2 mM β-Mercaptoethanol) and eluted with 5 CV of elution buffer (10 mM Tris-HCl pH 8.0, 250 mM imidazole, 1M NaCl, 2 mM β-Mercaptoethanol). HRV 3C protease was used to cleave the 6xHis-tag by incubation overnight at 4°C. The sample was dialyzed in equilibration buffer and the cleavage tag was separated from the protein by reverse IMAC. The protein-containing fractions were concentrated and purified further by size exclusion chromatography using a Hi-Load 16/600 Superdex 75 pg column (GE Healthcare). Peak fractions were concentrated and assessed for their purity (>95%) using SDS-PAGE.[33] The final protein fractions were dialyzed into a final buffer of 10 mM phosphate pH 6.9, 40 mM NaCl and 0.5 mM TCEP. Samples were stored in small aliquots at −80°C after snap freezing. Protein concentration was determined from the absorbance at 280 nm using theoretical extinction coefficient calculated by ProtParam ExPASy.[34]

RNA sample preparation

The RNA oligonucleotides (NNNNN, GAGCC, GAGAC, UAGAC, UGGAC, NANNN, NNANN, NNNAN, NNNNA, NCNNN, NNCNN, NNNCN, NNNNC, NGNNN, NNGNN, NNNGN, NNNNG, NUNNN, NNUNN, NNNUN, NNNNU) were purchased from Horizon Discovery and deprotected as advised by the manufacturer. Before use, the samples were lyophilised and resolubilized in 10 mM phosphate pH 6.9, 40 mM NaCl and 0.5 mM TCEP. RNA concentration was determined from the 260 nm absorbance of the sample.

NMR experiments

$^{13}C^{15}N$-labelled samples were prepared in a 90% H_2O/10% D_2O solvent ratio at a final concentration of 200 μM. NMR experiments were recorded at 25°C and 37°C on Bruker Avance and Varian Inova NMR spectrometers operating at 600, 700 and 800 MHz. $^{13}C^{15}N$ samples were used to acquire TROSY HNCA, ^{15}N-NOESY-HSQC, HNCA, TROSY[35] HNCACB[36] and HN(CO)CACB experiments. Spectra were processed using NMRPipe[29] and NMRDraw[37] and analyzed using Sparky[28] and CcpNMR Analysis.[26]

Scaffold independent analysis (SIA)

NMR-SIA was performed on FMRP KH1WT/KH2DD (WT) and FMRP KH1KK/KH2KK (RNA-AE) mutants. 50 μM stock protein was prepared in 40 mM NaCl, 10 mM phosphate pH 6.5, 0.5 TCEP, 0.002% sodium azide and RNasin Plus (Promega). RNA pools were added where required at a protein to RNA ratio of 1–4. Samples were transferred to 3 mm SampleJet NMR tubes (Sigma-Aldrich) and loaded into a Bruker Avance NMR spectrometer at 700 MHz using a Bruker Sample Jet loader. ^1H-^{15}N SOFAST-HMQC spectra[38] were recorded, processed and analyzed using NMRPipe and the weighted chemical shift perturbation (CSP) calculated according to the formula:

$$CSP = \sqrt{1/2 \cdot \left((\Delta\delta_{1H})^2 + (0.15 \cdot \Delta\delta_{15N})^2 \right)}$$

where δ_{1H} and δ_{15N} are the chemical shift differences of the ^1H and ^{15}N dimensions respectively. For each of the four pools, CSP values for each peak were normalized with respect to the highest shift observed for that pool. For each pool, the average normalized values across the subset of peaks are taken to provide a comparative score of binding preference, which is defined as the SIA score.

NMR binding assays

NMR titration experiments were performed using 50 μM of ^{15}N-labelled protein samples in 10 mM phosphate pH 6.9, 40 mM NaCl, and 0.5 mM TCEP, and titrated with unlabelled RNA oligonucleotides (NNNNN, GGCC, GAGAC, UAGAC, UGGAC) at protein-to-RNA molar ratios of 0, 0.5, 1, 2, 4, 6, 8, 10 and 12. Spectra were processed using Topspin 3.7 (Bruker) and a value for the ^1H and ^{15}N chemical shift changes was calculated for each residue by applying the equation:

$$CSP = \sqrt{1/2 \cdot \left((\Delta\delta_{1H})^2 + (0.15 \cdot \Delta\delta_{15N})^2 \right)}$$

The K_D values obtained were calculated using CcpNMR Analysis using the A(B + x-sqrt((B + x)ˆ2-4x) equation. The K_D value calculated per NMR binding assays corresponds to the average of the K_D's individual residues.

Thermal unfolding by circular dichroism (CD)

Thermal unfolding of FMRP KH12, FMRP KH1/KH2 (K299D/N300D) and FMRP KH1(T236K/H237K)/KH2(K299D/N300D) was monitored by CD, as previously described.[39] Experiments were performed on a Jasco J-815 spectropolarimeter equipped with CDF-426S

temperature-control system. Protein samples were prepared in 10 mM phosphate pH 6.9, 40 mM NaCl, 0.5 mM TCEP at 0.2 mg/mL. The solution was heated from 20°C to 95°C at a rate of 2°C per minute and the unfolding of the protein was monitored at 220 nm.

Sequence alignments

Primary sequences of RRM and KH containing proteins from the whole *H. sapiens* proteome were obtained in InterPro,[40] by searching the integrated signatures IPR000504 and IPR004087 respectively. Proteins were further considered only when annotated as 'reviewed' in the InterPro database. The sequences corresponding to the domains of interest were extracted and aligned with Clustal Omega[41] and alignment figures were generated using Jalview[30] using the CLUSTAL X conservation representation. Schematic representation of the RRM domain was obtained using BioRender.

QUANTIFICATION AND STATISTICAL ANALYSIS

Analysis explanation of the experiments can be found in the figure legends as well as in each subsection of the STAR Methods, including a definition of all the data.

Cell Reports Methods, Volume *3*

Supplemental information

Affinity-enhanced RNA-binding domains as tools to understand RNA recognition

Belén Chaves-Arquero, Katherine M. Collins, Giancarlo Abis, Geoff Kelly, Evangelos Christodoulou, Ian A. Taylor, and Andres Ramos

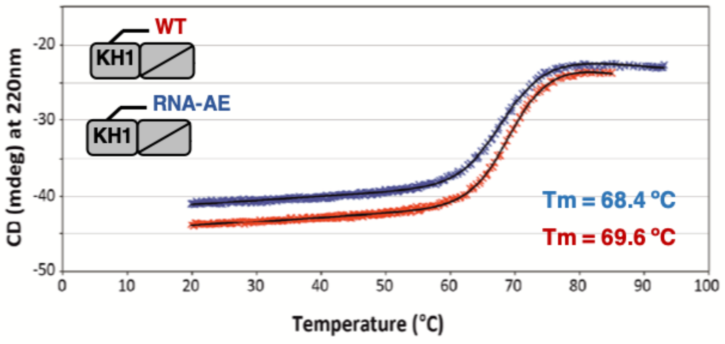

Figure S1: Stability of the affinity-enhanced FMRP KH12 GKKG mutant, related to Figure 1. Comparison of the thermal unfolding of FMRP KH12 WT (red) and RNA-AE (blue) monitored at 220 nm against temperature. Tm values are 69.9 ºC and 68.4 ºC respectively.

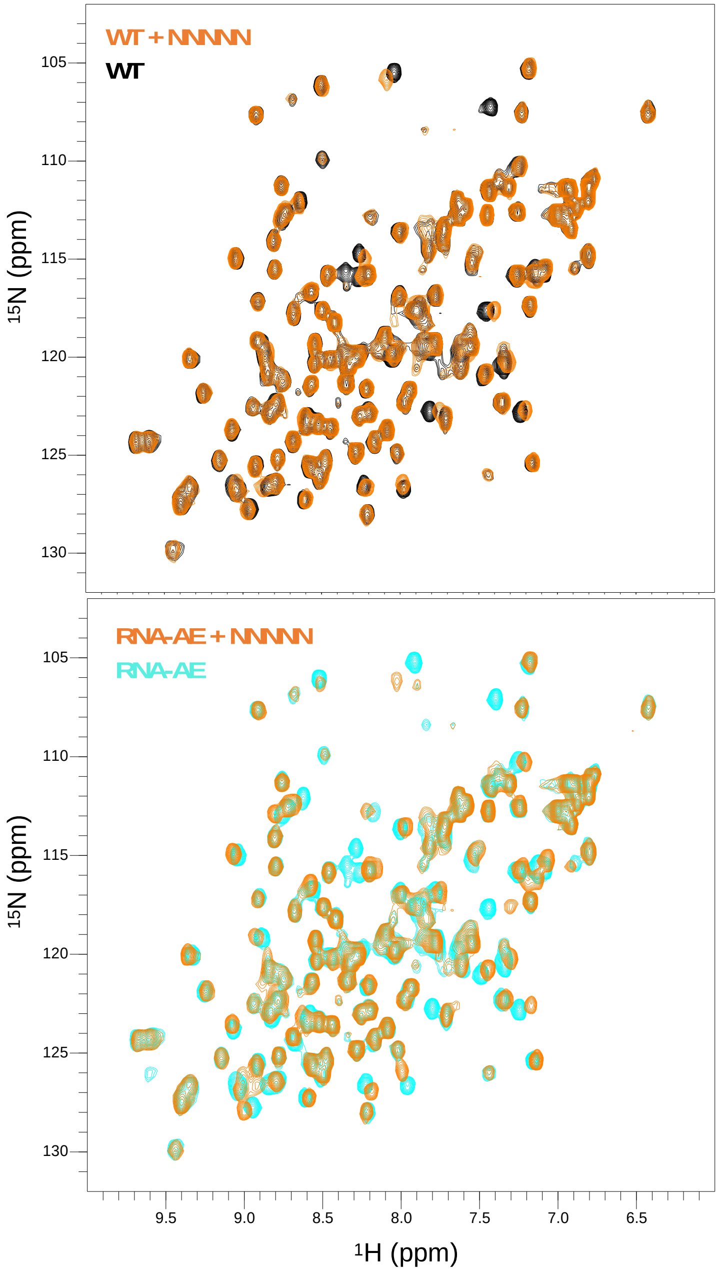

Figure S2: **RNA binding by the affinity-enhanced FMRP KH12 GKKG mutant, related to Figure 1.** Top: superimposition of ~1 hour ^{1}H-^{15}N HSQC spectra recorded on 50 μM FMRP WT free (black) and with NNNNN RNA at a 1:8 protein:RNA ratio (orange). Bottom: the equivalent superimposition is plotted for the spectra of FMRP RNA-AE free (light blue) and with NNNNN RNA at 1:8 protein:RNA ratio (orange).

A)

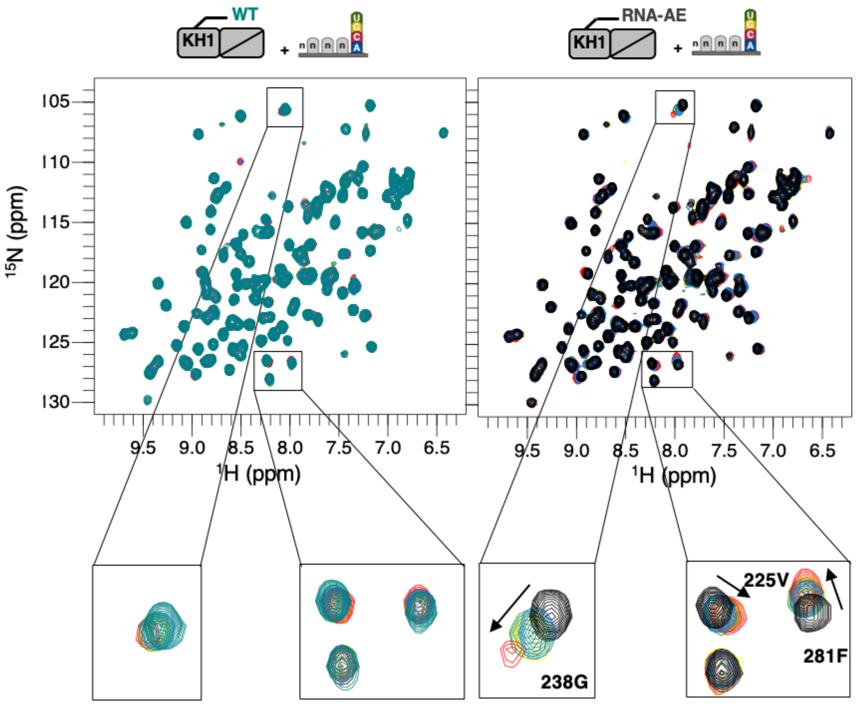

B)

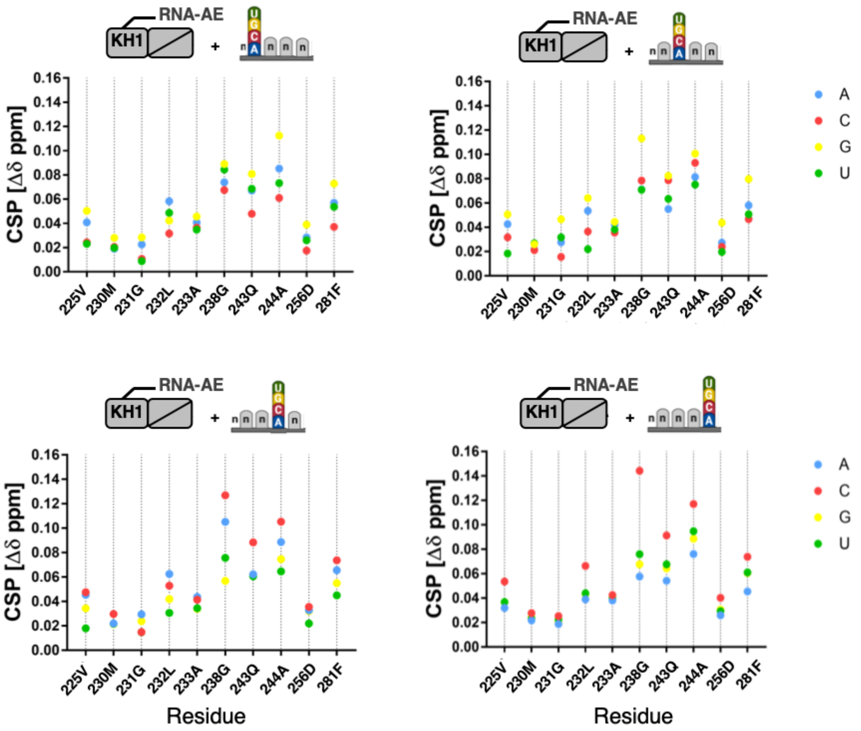

C)

	n X n n n	n n X n n	n n n X n	n n n n X
A	0.81	0.75	0.89	0.65
C	0.58	0.68	0.93	1.00
G	0.97	1.00	0.72	0.75
U	0.69	0.65	0.60	0.76

Figure S3. **Scaffold Independent Analysis (SIA) of the affinity-enhanced FMRP KH12 GKKG mutant, related to Figure 2.** The figure includes the Scaffold Independent Analysis (SIA) workflow, example data, and final scores. The SIA analysis provides a semi-quantitative, comparative estimate of the nucleobase preference of an RNA binding domain for each of the position of the bound oligonucleotide. (A) Superimposition of the ^{1}H-^{15}N HSQC spectra for the FMRP WT (left) and FMRP RNA-AE (right) free and when bound to each of the four quasi-randomized RNA pools which differ in the nucleobase to be examined (either A, C, G or U), for position 4 in this example. These superimpositions are used to measure the shift in peak position that report on the binding affinity of the four pools. Zoomed regions are for better comparison of the magnitude of the shifts observed in the 238G, 225V and 281F residues in both FMRP WT and FMRP RNA-AE spectra. The zoom-ins show that, while shifts in the wild type protein are very small, at the limit of the spectral resolution, shifts in the mutant can be measured with confidence and clear differences are visible. (B) Chemical shift changes (weighted average of ^{1}H and ^{15}N) of individual resonances of FMRP RNA-AE when binding to the four quasi-randomized RNA pools. For each peak, the values reported are normalized with respect to the highest shift value so that each peak contributes equally to the output. This is to ensure that any local difference due to the chemical nature of the bases to be scanned is more robustly averaged out across all the peaks, yielding final SIA scores depending on affinity. (C) Final SIA scores for the four positions.

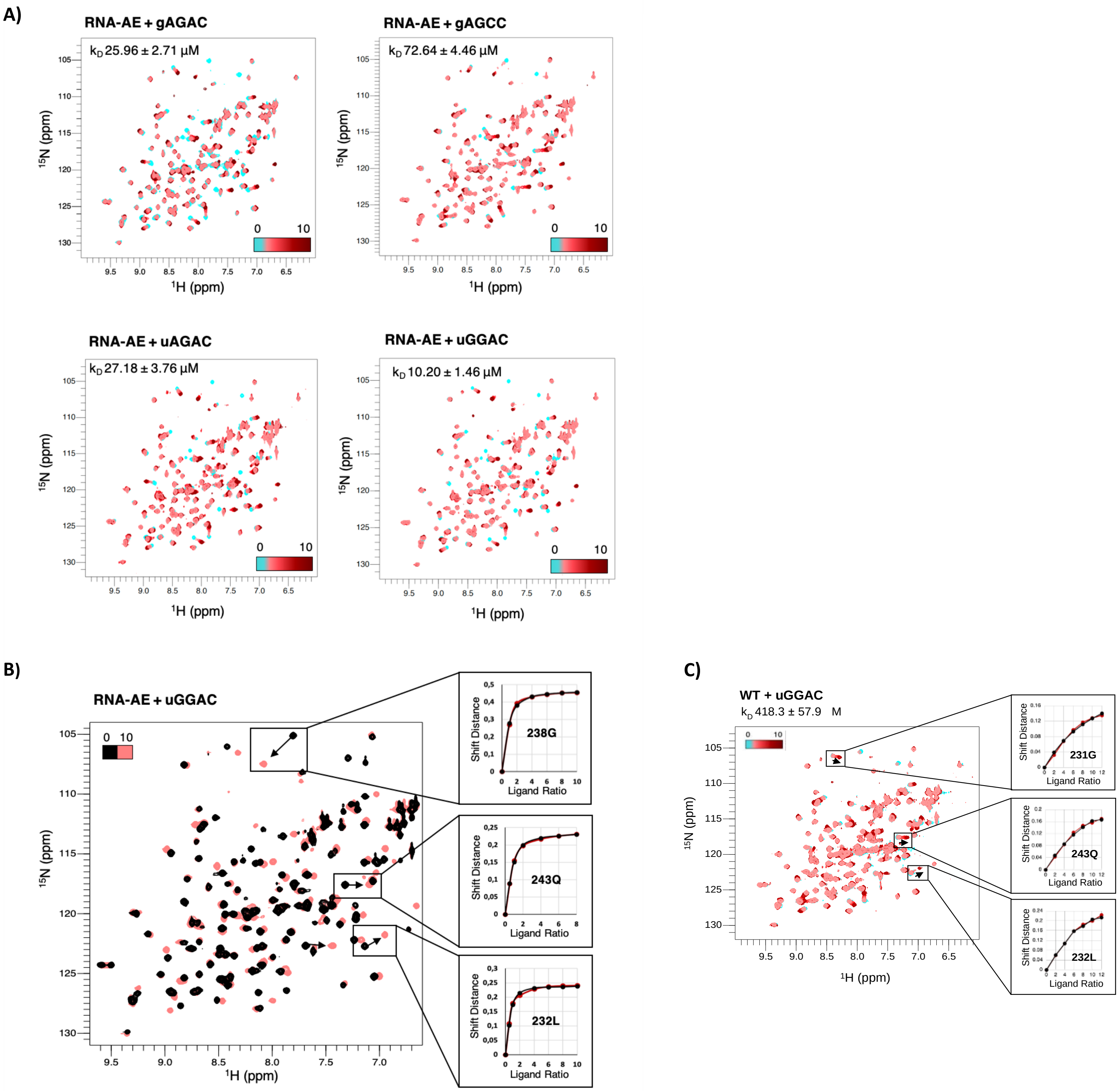
A)
RNA-AE + gAGAC
kD 25.96 ± 2.71 µM
RNA-AE + gAGCC
kD 72.64 ± 4.46 µM
RNA-AE + uAGAC
kD 27.18 ± 3.76 µM
RNA-AE + uGGAC
kD 10.20 ± 1.46 µM
0
10
15N (ppm)
1H (ppm)
B)
RNA-AE + uGGAC
0
10
238G
243Q
232L
Shift Distance
Ligand Ratio
15N (ppm)
1H (ppm)
C)
WT + uGGAC
kD 418.3 ± 57.9 M
231G
243Q
232L
Shift Distance
Ligand Ratio
15N (ppm)
1H (ppm)

Figure S4: **Affinity of FMRP KH12 wild type and affinity-enhanced for the specific sequences related to Figure 3.** RNA recognition by FMRP RNA-AE. (A) Overlaid ^{1}H-^{15}N HSQC spectra of FMRP RNA-AE with gAGAC, gAGCC, uAGAC and uGGAC at protein to RNA ratios of 1 to 0, 0.5, 1, 2, 4, 6, 8, 10. The spectra are coloured with increasingly dark red as we proceed along the titration. (B) Overlaid ^{1}H-^{15}N HSQC spectra of FMRP RNA-AE with uGGAC at protein to RNA ratio 1 to 10. The binding isotherm derived from the fit of chemical shift change against protein : RNA ratio is reported for three representative peaks, which are also displayed. Data points are coloured red, while the fit is in black. Fitting was performed using CcpNmr and the equation A(B+x-sqrt((B+x)^2-4x. Overall K_D values reported in the manuscript are an average of the ones obtained for the individual residues, and errors are reported. (C) Overlaid ^{1}H-^{15}N HSQC spectra of FMRP WT recorded during a titration with the preferred “uGGAC” sequence at protein-to-RNA ratios of 1 to 0, 0.5, 1, 2, 4, 6, 8, 10. The spectra are coloured with increasingly dark red as we proceed along the titration. The binding isotherms derived from the fit of chemical shift changes against protein:RNA ratios are reported for three representative peaks, the same used for the GKKG mutant in Figure S4b. In this plots, experimental data are in red, while the fit is in black. Fitting was performed using CcpNmr and the equation A(B+x-sqrt((B+x)^2-4x. The average K_D value and the errors are reported.
